# Olfactory Dysfunction as a Marker for Essential Hypertension in a Drug-Naive Adult Population: A Hospital-Based Study

**DOI:** 10.7759/cureus.41920

**Published:** 2023-07-15

**Authors:** Shria Datta, Kamlesh Jha, Abhimanyu Ganguly, Tribhuwan Kumar

**Affiliations:** 1 Physiology, All India Institute of Medical Sciences, Patna, IND

**Keywords:** olfactory receptor, indian smell identification test, olfactory threshold, olfactory dysfunction, drug naïve essential hypertension

## Abstract

Background and objective: Essential hypertension is a leading cause of cardiovascular morbidity worldwide, but its precise etiology remains unclear. Although its prevalence is high, there is no established predictor for the condition at an early age. Recent research has suggested that olfactory function may be associated with blood pressure regulation. This study sought to explore the association between olfactory function and essential hypertension.

Methods: Thirty middle-aged volunteers of both sexes with essential hypertension were recruited for the study along with 30 healthy control subjects matched for age and demographic characteristics. Participants completed a demographic questionnaire and then underwent olfactory function tests to assess odor threshold and identification using the Indian Smell Identification Test (InSIT). The researchers calculated a combined threshold-identification score for both groups and performed the statistical analysis.

Results: The study group showed significant olfactory scores in comparison to the control group participants. Control group showed a significantly higher mean combined olfactory score than the study population (p = 0.03). Significant negative correlation between systolic blood pressure and olfactory function (Pearson’s coefficient = -0.329, p = 0.011) and a similar significant negative correlation between diastolic blood pressure and olfactory function (Pearson’s coefficient = -0.252, p = 0.052) were the other observations. Gender differences did not account for any difference in the smell sense.

Conclusions: There may be a connection between olfactory function and blood pressure regulation in individuals with essential hypertension. However, further research is needed to better understand this association and to determine whether olfactory function could be used as a predictor or marker for hypertension.

## Introduction

Hypertension is one of the most common cardiovascular conditions with global prevalence affecting around 1.39 billion adults in the age group of 30-79 years, mostly (two-thirds) from low- and middle-income countries [1]. The seventh report of the Joint National Committee on the Prevention, Detection, Evaluation, and Treatment of High Blood Pressure (JNC 7) [2] classified hypertension into two stages: stage 1 hypertension (an average systolic BP of 140-159 mmHg or an average diastolic BP of 90-99 mmHg) and stage 2 hypertension (an average systolic BP of ≥160 mmHg or an average diastolic BP of ≥100 mmHg). It can be designated as essential hypertension when no clearly defined etiology is identified. When a definite secondary cause is evident, it is designated as secondary hypertension. Etiologically, essential hypertension is a conglomeration of diverse yet poorly defined conditions culminating in raised blood pressure. The possible etiological factor may include genetic, environmental, and behavioural factors. Around 90% of all hypertensive cases belong to the etiologically ill-defined group of essential hypertension [3].

The physiology of blood pressure control mechanisms is complex, and changes in blood pressure are compensated for by feedback mechanisms. However, when the balance between these mechanisms is disturbed, essential hypertension can develop [4]. This condition is often asymptomatic and can go undiagnosed until it causes severe outcomes such as heart attack, stroke, or chronic kidney disease. Early diagnosis is therefore crucial, and research into early indicators and treatments is necessary.

Olfaction is the sense of smell, and it involves the perception of chemical odors by the olfactory system. The olfactory transduction process begins with the contact of odorants with primary olfactory neurons, which then send signals to the secondary olfactory structures in the brain [5]. The impaired olfactory function has been linked to cognitive decline including Alzheimer's and Parkinson's diseases [6].

Recent attention has been drawn to olfactory changes as a potential diagnostic tool for COVID-19. Anosmia, or loss of smell, is a common symptom in COVID-19 patients and is caused by the virus's impact on olfactory epithelial cells that express angiotensin-converting enzyme 2 (ACE-2) receptors. These receptors are also present in renal olfactory receptors which release renin and play a crucial role in regulating blood pressure through the renin-angiotensin-aldosterone system (RAAS) mechanism [7-8].

This study aims to provide a crucial first step in understanding the relationship between olfaction and hypertension. By exploring the neural olfactory mechanism's link to cardiovascular pathology, further research can identify early indicators of hypertension and develop novel treatments. This study's results suggest that olfactory function may be a potential early indicator of hypertension, providing a new avenue for hypertension diagnosis and treatment.

## Materials and methods

It is an analytical cross-sectional study conceived to investigate the association of olfactory dysfunction with essential hypertension in the adult population of the region. Thirty newly diagnosed hypertensive volunteers in the age group of 30 to 50 years including both male and female have been recruited for the study after written informed consent. Thirty age and sex-matched healthy normotensive volunteers have been recruited as control group participants after due consent. It was a quasi-purposive sampling with the recruitment of volunteers visiting the Institute Medicine OPD within the reference period (two months) and consenting to the study. A prior ethical clearance has been taken from the Institutional Ethical Committee of the All India Institutes of Medical Sciences, Patna (AIIMS/Pat/IEC/2022/913).

Participants diagnosed with essential hypertension as per JNC 7 recommendations were included in the study group. Participants with systolic blood pressure less than 140 mmHg and diastolic blood pressure less than 90 mm Hg (as per JNC 7 recommendations) have been included in the control group. The participants with a history of major acute or chronic illness, a history of major neurological diseases like Alzheimer’s, and Parkinson’s, etc, those suffering from any disorder known to cause alternation in the sense of smell, having a recent history of COVID-19 positivity (in the last six months), or those with any known causes for hypertension (secondary hypertension) like chronic kidney disease, obstructive sleep apnoea, hormonal abnormality history of long term medications like NSAIDs, antidepressants or birth control pills, or chronic alcoholic participants were excluded from the study.

Methodology

After the recruitment, demographic information was collected using a pre-designed questionnaire. The questionnaire was used to gather participant data such as age, smoking status, alcohol intake, history of hypertension, any smell-related disorder along with current medications, etc. Further two types of olfactory tests have been performed using the olfactory threshold test (OTT) and Indian smell identification test (InSIT). The olfactory function tests were performed to evaluate the odor threshold of the participant in which the minimum concentration of the odorant necessary for a smell to be perceived by a person was determined; olfactory discrimination test was performed to assess a person's ability to differentiate one smell from another. A combined threshold-identification score was calculated for both test and control groups to understand the overall status of the olfactory function of the participant.

OTT

Test odorant 1-butanol was employed as the material for the OTT. 1-butanol is commonly used as it is low in toxicity, colourless, water-soluble, readily available in high purity, and has a neutral odor quality [9]. Because of these attributes, it has been widely accepted as a reference odorant in various applied settings. The test kit contained five glass bottles each containing ~20 ml of test solution which were labelled as solutions one to five, and another identical glass bottle filled with ~20 ml of sterile water. The 1-butanol solution was diluted by successive factors of two; the highest concentration was 1:2, designated as solution one while the lowest concentration was 1:32, designated as solution five. Participants received two bottles at a time, one with sterile water and one with odorant (solutions one to five). The test was carried out with the weakest solution in an ascending order of concentration to avoid desensitization. The method of ascending limits was used. The lowest concentration of odorant that the patient identified was taken as the threshold limit. If the solution was correctly identified, one point was awarded else zero was scored [10].

InSIT

InSIT [11] was employed in which the odorants used were the essence of 10 commonly used items. The choice of odorants was based on familiarity with day-to-day life. The essence of cardamom, lemon, mango, coffee, rose, garlic, Dettol, mustard oil, sandalwood, and asafoetida in 20 ml air-tight bottles were used. The bottles containing test materials were placed one by one, in front of the nostrils of the participants (at a distance of 1 cm from the nostril) with the lid open for three seconds; the patient was asked to sniff normally without any force, with the other nostril closed. To prevent olfactory desensitization, an interval of at least 30 seconds between successive presentations was maintained. The process was repeated for the other nostril also. The subject was asked to identify the smell. The response was taken and scored 1 for correct and 0 for wrong response. A combined olfactory score was computed by adding the total positive scores of both the OTT and InSIT.

Data analysis

For the purpose of analysis , a P value of less than 0.05 was taken as significance level. Data analysis was done using SPSS version 22 (IBM Corp., Armonk, NY). The data has been tested for the normality of distribution using Shapiro-Wilk test, Q-Q plot, and box plot methods. The normally distributed interval data has been expressed as a mean with 95% CI and analysed using parametric tests. The non-normally distributed observations have been expressed as median with interquartile range and have been analysed using non-parametric tests. Pearson’s correlation analyses were performed to evaluate the relationship between combined threshold-identification score and systolic blood pressure, diastolic blood pressure, pulse pressure, and mean arterial pressure.

## Results

The demographic and blood pressure findings of the control and study group population is summarized in Table 1.

**Table 1:** The demographic and cardiovascular parameters of the study participants mm Hg: Millimeters of Mercury; bpm: beats per minute; SD: standard deviation.

Subject characteristics	Test Group (mean + SD)	Control Group (mean + SD)	P value
Age (years)	42.6 ± 6.05	42.6 ± 8.08	1.00
Height (cm)	162 ± 8.94	159 ± 6.32	0.08
Weight (kg)	66.5 ± 16.7	60.4 ± 10.5	0.094
Smoking (%)	37%	13%	0.037
Alcohol intake (%)	3%	10%	0.309
Systolic Blood Pressure (mm Hg)	154 ± 18.9	117 ± 8.4	<0.001
Diastolic Blood Pressure (mm Hg)	95 ± 12.1	73.3± 5.94	<0.001
Pulse (bpm)	98.6 ± 19.4	89.8 ± 12.9	0.043
Mean Arterial Pressure (mm Hg)	115 ± 13.1	88 ± 5.16	<0.001
Pulse Pressure (mm Hg)	59.3 ± 14.5	44.2 ± 9.58	<0.001

**Figure 1:**
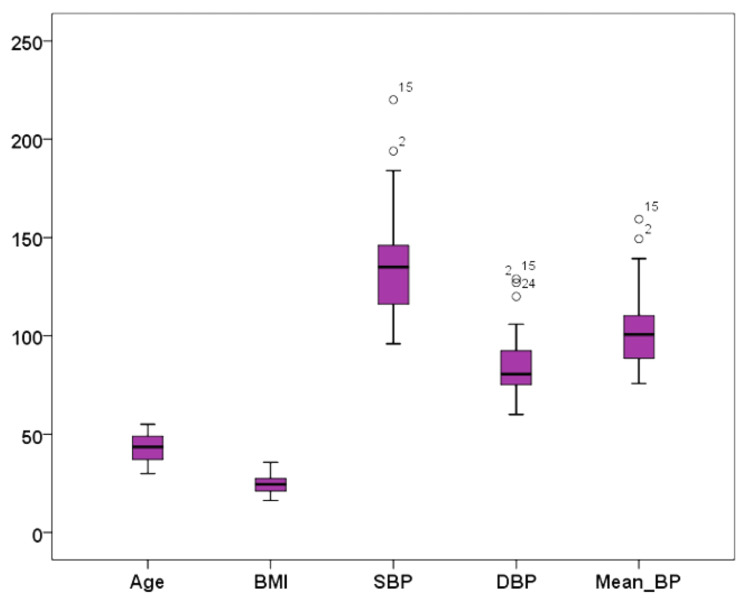
Box plot showing the distribution of variables BMI: body mass index; SBP: systolic blood pressure; DBP: diastolic blood pressure.

The variables were tested for the normality of distribution using box plots, Q-Q plots, and Kolmogorov- Smirnov test with Lilliefors significance correction and were found normally distributed for most of the measured variables except for the mean BP (Figure 1).

The mean combined olfactory scores, computed by combining the composite OTT and composite InSIT, for the test and control group were 9.13 ± 2.21 and 10.7± 1.62, respectively. The result of the olfactory function test is presented in Table 2.

**Table 2:** Comparison of odor test values in hypertensive and normotensive participants OTT: olfactory threshold test; InSIT: Indian smell identification test.

Variables	Normotensive Gr. (control)Mean + SD (n= 30)	Hypertensive Gr. (Case) mean + SD (n=30)	95% CI	t Value	P Value
Composite OTT	4.53 + 0.51	4.23 + 0.82	-0.053 to 0.65	1.71	0.09
Composite InSIT	6.13 + 1.38	4.9 + 1.9	0.35 to 2.1	2.8	0.007
Combined Olfactory score	10.7 + 1.6	9.1 + 2.21	0.53 to 2.54	3.04	0.03

**Figure 2:**
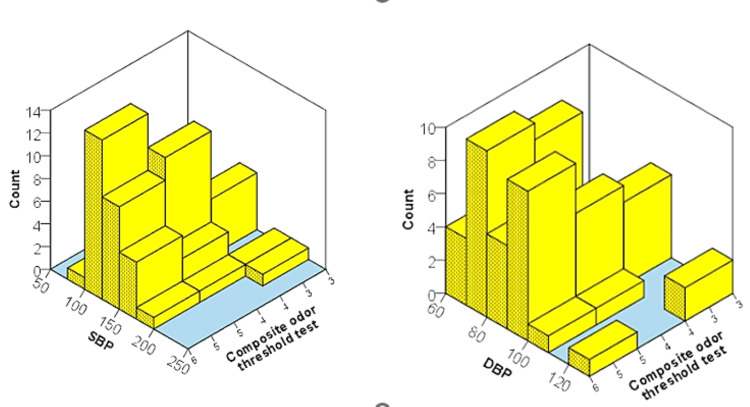
The 3D graph showing the relationship of SBP and DBP with Composite odor threshold scores SBP: systolic blood pressure; DBP: diastolic blood pressure.

The Independent sample t-tests showed that the study group had lower mean OTT, InSIT, and combined olfactory scores in comparison to the control group participants though the difference was not statistically significant for the olfactory threshold test. Participants with lower blood pressure scored more in the combined odor threshold test in comparison to those with higher blood pressure (Figure 2).

The correlation study between various cardiovascular and demographic parameters recorded in the study using Pearson’s correlation test shows that both InSIT and OTT scores have a negative correlation with systolic blood pressure, diastolic blood pressure, and mean BP. The age of the participants has shown a statistically significant negative relationship with InSIT score but not with OTT. BMI appears to be positively correlated with the OTT score (Table 3).

**Table 3:** The correlation between various study parameters and olfactory scores * significant negative or positive correlation. SBP: systolic blood pressure; DBP: diastolic blood pressure; OTT: olfactory threshold test; InSIT: Indian smell identification test.

Variables	SBP	DBP	Mean BP	Age	BMI	Composite InSIT	Composite OTT
SBP	1	0.82	0.94	0.17	0.05	-0.24*	-0.33*
DBP		1	0.96	0.04	-0.07	-0.16	-0.32*
Mean_BP			1	0.1	-0.01	-0.21	-0.34*
Age				1	-0.1	-0.27*	-0.2
BMI					1	0.04	0.25*
Composite InSIT						1	0.25
Composite OTT							1

The chi-square test showed that out of 10 items used in the InSIT, the essence of rose could be perceived less efficiently by hypertensive individuals in comparison to non-hypertensive participants ( χ2= 6.37; p= 0.01) (Table 4).

**Table 4:** InSIT chi-squired values for various odorants InSIT: Indian smell identification test.

InSIT test Object	Cardamom	Asafetida	Lemon	Mango	Coffee	Rose	Garlic	Detol	Mustard oil	Sandalwood
Chi-square value	2.58	0.05	0.05	0.47	0.05	6.37	2.58	0.05	0.47	0.05
p Value	0.1	0.8	0.8	0.5	0.8	0.01	0.1	0.8	0.5	0.8

## Discussion

This study found a statistically significant association between olfactory function and hypertension. The subjects with hypertension showed a higher olfactory threshold for the serial dilutions of 1-butanol used for the purpose, in comparison to those with normal blood pressure, though the difference was not statistically significant (p=0.09). Both systolic as well diastolic pressure values showed a negative correlation with the odor threshold scores. A similar finding was also observed in InSIT. InSIT scores showed a better correlation with systolic blood pressure than diastolic or mean blood pressure. The combined olfactory score showed significantly higher values for normotensive individuals in comparison to hypertensive individuals. In this study, age was found to have a negative correlation with InSIT score but it did not show any correlation with OTT. The findings agree with many previous studies [12-14].

Blood pressure has a well-established relationship with cardiovascular morbidity including ischemic heart disease, heart failure, cardiac remodelling, etc. Besides, it is also implicated in the pathogenesis of stroke, reno-vascular pathophysiology, and many other systemic disorders with hypertensive angiopathies.

The altered smell perception in hypertension may be associated with some pathogenic mechanism, commonly associated with both the olfactory as well as blood pressure control pathways. One possible mechanism could be the shared olfactory receptor 78 (Olfr78) present in both the olfactory apparatus and various extra nasal sites including the kidneys. Another mechanism linking smell and taste sensation with hypertensive disorders has been proposed by Pluznick et al. (2013) suggesting that the short-chain fatty acids produced by the gut flora play an important role in the upregulation of renin causing raised blood pressure [8].

A study utilizing RNA sequencing on human tissues discovered that olfactory receptors exist in various locations outside the nasal region such as sperm, muscle, skin, adipose tissue, and the gastrointestinal tract, etc. These receptors serve diverse functions ranging from facilitating cell migration and motility to the release of hormones. Interestingly, the kidneys (traditionally recognized as an organ for filtration and excretion) also exhibit sensory characteristics. Within the kidney, the renal vasculature and tubular epithelial cells assume the role of monitoring the blood and ultrafiltrate composition, regulating processes such as filtration, reabsorption, and secretion. Notably, the human kidney contains some 13 distinct olfactory receptors, believed to contribute significantly to maintaining renal homeostasis [7]. The olfactory receptors present in the nose and the one present in the kidney share a similar signalling pathway using trimeric G proteins as evidenced by the studies using AC3 knockout mouse, delineating its role in renal homeostasis. Hypothetically, the same signalling pathway may also be involved in the activation of juxta glomerular apparatus (JGA) cells causing the activation of the renin-angiotensin-aldosterone mechanism causing hypertension.

Previous studies have shown that cardiovascular disorders are intimately associated with olfaction [15]. A longitudinal study by Thiebaud et al. stated that subclinical atherosclerosis was found to be significantly associated with a decline in olfaction. The study further claimed that olfaction atherosclerosis may be used as a predictor for the decline in olfaction with age. An experiment conducted on mice revealed a notable decrease in olfactory neurons and their axonal projections following exposure to a fatty diet. Moreover, there was a decline in olfactory discrimination ability. The authors concluded that the consumption of a high-fat diet induced a pro-inflammatory state, potentially leading to an impaired sense of smell. This outcome was attributed to the increased presence of macrophages and neuronal death, resulting in reduced connections between the olfactory epithelium and the olfactory bulb [16].

The sample size was one of the limitations of the study, Further research may be needed with a bigger sample size to conclusively establish or refute the olfactory dysfunction as a marker for essential hypertension. Despite its limitations, the present work attempts to highlight the importance of olfactory function in the pathophysiology of conditions like hypertension.

## Conclusions

The study demonstrates that subjects with hypertension had lower smell perception as measured by the combined olfactory score and InSIT. The subjects with hypertension showed a lower mean olfactory threshold in comparison to the normotensive individuals but the difference was not statistically significant. Further, systolic blood pressure, diastolic blood pressure, and mean BP of the participants showed a negative correlation with the olfactory function but the strength of association appears to be weak. Out of the common substances of the InSIT, the smell of rose showed some association with the trait of essential hypertension which may require further studies in the area for a conclusive remark.
